# Internalizing Characteristics in Adolescents with Non-Cardiac Chest Pain: A Cross-Sectional Study from Turkey

**DOI:** 10.3390/children13020265

**Published:** 2026-02-13

**Authors:** Veli Yıldırım, Fatih Battal, Recep Dokuyucu

**Affiliations:** 1 Department of Child Psychiatry, Special Clinic, Yenişehir 33110, Mersin, Turkey; 2 Department of Pediatrics, Faculty of Medicine, Çanakkale Onsekiz Mart University, Çanakkale 17020, Turkey; 3 Department of Physiology, Medical Specialization Training Center (TUSMER), Ankara 06230, Turkey

**Keywords:** adolescents, anxiety, depression, internalizing symptoms, maternal age, non-cardiac chest pain, psychosocial stress

## Abstract

**Highlights:**

**What are the main findings?**

- Adolescents with non-cardiac chest pain show significantly elevated internalizing symptoms, particularly anxiety, depression, and overall psychological distress.
- Female gender and a prior history of psychosocial symptoms are associated with higher anxiety and symptom burden, while higher maternal age is linked to lower perceived stress.

**What are the implications of the main findings?**

- Non-cardiac chest pain in adolescents should be considered a potential somatic marker of underlying psychological distress rather than a purely benign physical complaint.
- Routine psychological screening may improve early identification and management of at-risk adolescents, reducing unnecessary medical investigations and healthcare utilization.

**Abstract:**

Background/Objectives: This study aimed to investigate the internalizing characteristics, including anxiety and depressive symptoms, in adolescents presenting with non-cardiac chest pain (NCCP), and to explore the effects of sociodemographic variables and prior psychosocial experiences on psychological distress. Methods: This cross-sectional study was conducted in Turkey and included 128 adolescents aged 10–18 years (57.0% female, 43.0% male) who presented to pediatric cardiology or general pediatric outpatient clinics. The Children’s Depression Inventory (CDI), State–Trait Anxiety Inventory for Children (STAI-C), Screen for Child Anxiety Related Emotional Disorders (SCARED), Brief Symptom Inventory (BSI), and the Social Support Appraisals Scale for Children (SSAS-C). Sociodemographic variables and prior psychosocial symptom history were also recorded. Results: Adolescents with non-cardiac chest pain exhibited elevated anxiety and psychological distress compared to controls. Female participants demonstrated higher levels of stress, anxiety, and overall psychological symptom burden than males. Higher maternal age was associated with lower perceived stress, while a prior history of psychosocial symptoms was linked to increased anxiety and global psychological distress. Participants with a history of psychosocial symptoms had higher anxiety (*p* = 0.027) and BSI (*p* = 0.004) scores. Significant positive correlations were found between anxiety, depression, obsessive–compulsive symptoms, and the BSI total score (r values ranging from 0.718 to 0.892). Conclusions: Adolescents with NCCP exhibit significant internalizing symptoms, particularly anxiety and depression. Female gender and prior psychosocial stressors were associated with elevated symptom scores. Maternal age may have a buffering effect on adolescent stress levels. These findings underscore the importance of integrating psychological screening into the evaluation of chest pain in adolescents to enable early identification and intervention.

## 1. Introduction

Chest pain is a common presenting complaint in pediatric and adolescent populations and frequently leads to emergency department visits and specialist referrals. Unlike adults, where chest pain often raises concern for cardiac pathology, the majority of cases in children and adolescents are attributable to non-cardiac etiologies, including musculoskeletal, gastrointestinal, and functional causes [1]. Nevertheless, non-cardiac chest pain (NCCP) remains a distressing symptom for adolescents and their families, often resulting in repeated medical evaluations, school absenteeism, and increased healthcare utilization.

Growing evidence suggests that unexplained or recurrent somatic symptoms in youth may represent manifestations of underlying psychological distress rather than isolated physical conditions [2,3]. Internalizing symptoms—particularly anxiety and depression—have been consistently associated with functional somatic complaints such as abdominal pain, headaches, and chest pain [4,5,6]. Adolescents experiencing NCCP have been shown to exhibit higher levels of emotional distress, impaired psychosocial functioning, and reduced quality of life compared to their healthy peers [7,8].

From a theoretical perspective, NCCP in adolescence can be conceptualized within a biopsychosocial framework, in which biological vulnerability, psychological processes (e.g., anxiety sensitivity and emotion regulation difficulties), and social–familial factors interact to shape symptom perception and expression [6,9]. Somatization models further propose that emotional distress may be communicated through physical symptoms during developmental periods when verbal emotional expression is limited or stigmatized [10,11]. This framework is particularly relevant in adolescence, a developmental stage characterized by heightened emotional reactivity and sensitivity to social stressors [12]. Although non-cardiac chest pain shares certain features with broader functional somatic syndromes, it represents a clinically distinct presentation characterized by acute symptom perception, heightened cardiac-related threat appraisal, and frequent medical referral. Unlike chronic multisite somatic syndromes, chest pain in adolescents often prompts immediate concern for serious organic pathology, which may amplify anxiety and symptom vigilance. Therefore, conceptualizing non-cardiac chest pain as a specific functional symptom—rather than as part of a generalized somatization spectrum—is essential for understanding its unique psychological correlates and clinical implications.

Previous studies have demonstrated that internalizing symptoms are more prevalent among adolescent girls, suggesting gender-related differences in emotional processing and symptom expression [3,13]. Additionally, family-related factors, including parental psychological characteristics and parenting dynamics, have been implicated in adolescent stress regulation and emotional well-being [14,15]. However, the influence of parental demographic variables—such as maternal and paternal age or education level—on psychological distress in adolescents with NCCP remains insufficiently explored [8,11,16,17,18].

Importantly, much of the existing literature has relied on single-scale assessments or has focused on isolated psychological outcomes, limiting a comprehensive understanding of the multidimensional nature of internalizing symptoms in adolescents with NCCP [7,19]. Moreover, few studies have simultaneously examined anxiety, depressive symptoms, broader psychological distress, and prior psychosocial vulnerability within the same cohort using multiple validated instruments [9,20].

Based on the existing evidence, we hypothesized that adolescents presenting with non-cardiac chest pain would exhibit elevated levels of anxiety, depressive symptoms, and overall psychological distress [7,10]. We further hypothesized that female adolescents would demonstrate higher internalizing symptom scores compared to their male counterparts, reflecting previously reported gender-related differences in emotional processing and symptom expression [12,13]. In addition, we anticipated that a prior history of psychosocial symptoms would be associated with greater psychological distress and symptom burden [21,22]. Finally, we hypothesized that parental factors—particularly maternal characteristics—would be associated with adolescents’ perceived stress levels and internalizing symptoms, consistent with earlier findings highlighting the role of maternal influences on adolescent emotional regulation [14,15,23].

Therefore, the present study aimed to provide a comprehensive evaluation of internalizing characteristics in adolescents presenting with non-cardiac chest pain using multiple validated psychometric instruments. By examining gender differences, parental demographic factors, and prior psychosocial symptom history, this study seeks to address important gaps in the literature and to clarify the psychological profile associated with NCCP in adolescence.

## 2. Materials and Methods

### 2.1. Study Design and Participants

This cross-sectional study was conducted between March 2016 and June 2019 at the Pediatric Cardiology and General Pediatrics Outpatient Clinics of Çanakkale Onsekiz Mart University. The study population consisted of adolescents aged between 10 and 18 years who presented with complaints of chest pain.

Participation in the study was entirely voluntary. Written informed consent was obtained from all adolescents, as well as from their parents or legal guardians prior to enrollment. All data were collected anonymously, and participants were assigned unique identification codes to ensure confidentiality and prevent personal identification. Adolescents aged 10–18 years who were able to understand and complete the study questionnaires and who agreed to participate were eligible for inclusion. Exclusion criteria included the presence of organic cardiac pathology identified during clinical evaluation, chronic or severe medical conditions that could impair functional capacity, diagnosed endocrine disorders involving the hypothalamic–pituitary–adrenal axis or adrenal glands, and a history of clinically diagnosed psychiatric disorders requiring ongoing treatment.

The control group consisted of adolescents attending the general pediatric outpatient clinic for routine health evaluations or minor, non-chronic complaints. These participants had no current or past history of chest pain and no known cardiac disease. In addition, none had a prior diagnosis of a psychiatric disorder or were receiving psychological or psychiatric treatment at the time of assessment. Adolescents who reported clinically significant psychological complaints requiring immediate referral were not included in the control group.

Parental age, education level, and employment status were collected as readily obtainable sociodemographic indicators reflecting the familial and caregiving context of the adolescents. These variables were selected because they are commonly used in clinical and epidemiological research as proxy measures related to parental experience, cognitive resources, and daily caregiving environment, all of which may influence adolescents’ stress perception and emotional regulation. A composite measure of socioeconomic status was not included, as reliable income-based data were not consistently available for all participants.

All participants were given a set of forms, including a Personal Information Form, and the following validated psychometric instruments. Stationery costs were covered by the researchers, and the completion time was approximately 20 min per participant.

### 2.2. Measurement Tools

Children’s Depression Inventory (CDI): Developed by Kovacs in 1981 [24], this scale evaluates depressive symptoms in children aged 6–17 years. It consists of 27 items, each scored from 0 to 2, with a maximum total score of 54. Each item is scored on a 3-point scale ranging from 0 to 2 (0 = absence of the symptom, 1 = moderate symptom, 2 = severe symptom), with higher total scores indicating greater depressive symptom severity. A score of 19 or above is considered indicative of significant depressive symptoms. The Turkish validity and reliability study was conducted in 1991 [25].

State–Trait Anxiety Inventory for Children (STAI-C): Developed by Spielberger et al., this inventory consists of two 20-item subscales assessing state anxiety (STAI-I) and trait anxiety (STAI-II). The STAI-C consists of two 20-item subscales assessing state and trait anxiety. Items are rated on a 4-point Likert scale, with response options ranging from 1 (almost never) to 4 (almost always). Higher scores indicate greater anxiety levels. A score of ≥45 indicates clinically significant anxiety [26]. The Turkish version was validated in 1985 [27].

Screen for Child Anxiety Related Emotional Disorders (SCARED): Developed by Birmaher et al. in 1997 [28], this 41-item Likert-type scale assesses anxiety symptoms in children aged 8–18. Items are scored on a 3-point Likert scale (0 = not true or hardly ever true, 1 = sometimes true, 2 = very true or often true), and the total scores range from 20 to 80. Scores range from 0 to 82, with higher scores indicating higher anxiety levels. The Turkish validation study was completed by Yalın Sapmaz [29].

Brief Symptom Inventory (BSI): A 53-item self-report scale derived from the SCL-90-R, the BSI assesses a broad range of psychological symptoms across 9 subscales, including depression, anxiety, somatization, and hostility. Each item is rated on a 5-point Likert scale ranging from 0 (not at all) to 4 (extremely). Higher scores reflect greater symptom burden [30]. Turkish adaptation and validation for adolescents were completed in 2002 in a sample of 597 adolescents [31].

Social Support Appraisals Scale for Children (SSAS-C): Originally developed by Dubow and Ullman (1989) [32], this scale evaluates children’s perceived social support from family, friends, and teachers. Items are rated on a 5-point Likert scale ranging from 1 (strongly disagree) to 5 (strongly agree). Higher total scores reflect stronger perceived social support. Turkish validation was carried out in 2007 by Ilgın Gökler in a cohort of children aged 9–17 years [33].

In the study, internal consistency was assessed using Cronbach’s alpha coefficients. The Children’s Depression Inventory demonstrated good internal consistency (Cronbach’s α = 0.84). The State–Trait Anxiety Inventory for Children showed satisfactory reliability for both the state anxiety subscale (Cronbach’s α = 0.88) and the trait anxiety subscale (Cronbach’s α = 0.86). The Screen for Child Anxiety Related Emotional Disorders exhibited excellent internal consistency (Cronbach’s α = 0.91). The Brief Symptom Inventory total score demonstrated high internal consistency (Cronbach’s α = 0.93). The Social Support Appraisals Scale for Children also showed good reliability in the present sample (Cronbach’s α = 0.89). All psychological data in the present study were obtained using self-report questionnaires completed by the adolescents.

### 2.3. Statistical Analysis

All statistical analyses were performed using SPSS version 27.0 (IBM Corp., Armonk, NY, USA). Descriptive statistics were expressed as frequencies, percentages, means, standard deviations, medians, and minimum–maximum values. The normality of continuous variables was initially assessed using both the Shapiro–Wilk and Kolmogorov–Smirnov tests. Given its higher sensitivity for small to moderate sample sizes, statistical decisions regarding data distribution were primarily based on the Shapiro–Wilk test. For normally distributed variables, comparisons between the two groups were made using the Independent Samples *t*-test. For non-normally distributed variables, the Mann–Whitney U test was used. Spearman’s correlation analysis was applied to examine the relationship between continuous non-parametric variables. An a priori sample size calculation was not performed, as the study was based on a convenience sample of eligible adolescents presenting during the study period. A *p*-value < 0.05 was considered statistically significant.

## 3. Results

Demographic characteristics of the study group are shown in Table 1. In this study, 128 adolescents were included, of whom 57.0% (n = 73) were girls and 43.0% (n = 55) were boys. Regarding the participants’ education, nearly half (48.4%) were in middle school, followed by high school students (39.1%). Most mothers were married (87.5%) and the majority had completed only primary school education (50.0%). About two-thirds of the mothers were housewives (64.8%). As for fathers, the most common education level was also primary school (37.9%), and the largest occupational group was self-employed (39.7%). In the study group, 10.2% (n = 13) were in 4th grade, 14.8% (n = 19) in 5th grade, 12.5% (n = 16) in 6th grade, 13.3% (n = 17) in 7th grade, 9.4% (n = 12) in 8th grade, 12.5% (n = 16) in 9th grade, 10.9% (n = 14) in 10th grade, 10.9% (n = 14) in 11th grade, and 5.5% (n = 7) in 12th grade (Table 1).

The analysis of responses to the Stress Factors Scale revealed that the most frequently reported fear among participants was the fear of experiencing pain (57.0%), followed by fear of being separated from a parent (53.9%) and fear of getting an injection (41.4%). Other common stress factors included fear of being forced to provide personal information (34.4%), fear of talking to strangers (32.0%), and fear of undressing during a medical examination (32.8%). Less frequently reported fears were fear of being hospitalized (29.7%), fear of being questioned by parents (25.0%), and fear of seeing blood (21.9%) (Table 2).

Among the predisposing factors evaluated, the most commonly reported was having a very close friend (82.8%), suggesting strong peer bonds among participants. Other notable factors included changing schools within the last month (17.2%), skipping school or engaging in disciplinary actions (15.6%), and changing classes within the last six months (14.8%). Less frequent but potentially impactful life events included moving house in the last six months (14.1%), experiencing a death in the family within the past two years (10.9%), the birth of a new sibling in the last year (8.6%), and parental divorce in the past two years (7.8%) (Table 3).

Scores received by participants from Psychiatric Scales are shown in Table 4. Adolescents demonstrated moderate to high levels of anxiety and psychological distress across the applied scales. Anxiety-related measures and global psychological symptom burden were notably elevated in the study sample (Table 4).

Distribution of Psychiatric Scale scores according to maternal education level is shown in Table 5. When the psychiatric scale scores of adolescents were compared based on their mothers’ education level, no statistically significant differences were observed between the two groups (*p* > 0.05 for all comparisons). The mean score for stress-related factors was slightly higher in adolescents whose mothers had primary school education or below (mean = 3.5 ± 2.3) compared to those whose mothers had high school education or above (mean = 2.8 ± 2.2); however, this difference was not statistically significant (*p* = 0.148). Similarly, predisposing factors did not show a significant difference between groups (mean = 1.8 ± 1.4 vs. 1.5 ± 0.9, *p* = 0.455). The Anxiety Disorders Screening Scale scores were comparable between the groups (28.5 ± 9.9 vs. 30.5 ± 14.0, *p* = 0.445), as were the scores for the State Anxiety Inventory (42.8 ± 7.2 vs. 42.9 ± 6.9, *p* = 0.567) and the Trait Anxiety Inventory (44.7 ± 6.1 vs. 45.7 ± 7.4, *p* = 0.431). Finally, total scores from the Brief Symptom Inventory were also similar across groups (37.9 ± 35.9 vs. 36.9 ± 31.9, *p* = 0.960) (Table 5).

Distribution of Psychiatric Scale Scores by gender is shown in Table 6. Girls had significantly higher scores than boys in the stress factors scale (mean = 3.86 ± 2.0 vs. 2.50 ± 2.0; *p* = 0.001). Similarly, girls scored significantly higher on the Anxiety Disorders Screening Scale (32.79 ± 13.6 vs. 25.31 ± 7.9; *p* < 0.001). Furthermore, total scores from the Brief Symptom Inventory (BSI) were notably higher among girls (45.9 ± 31.2) than boys (26.9 ± 27.9), with this difference also being statistically significant (*p* = 0.001). No significant gender differences were found in state anxiety (*p* = 0.645), trait anxiety (*p* = 0.075), or predisposing factors (*p* = 0.277) (Table 6).

Correlation between maternal age and Psychiatric Scales is shown in Table 7. A statistically significant negative correlation was found between maternal age and Stress Factors scores (r = −0.259, *p* = 0.003). No statistically significant correlations were found between maternal age and other Psychiatric Scales, including Predisposing Factors, Anxiety Disorders Screening Scale, State and Trait Anxiety Inventories, or the Brief Symptom Inventory total score (*p* > 0.05 for all) (Table 7).

Correlation between paternal age and Psychiatric Scales is shown in Table 8. No statistically significant correlations were found between paternal age and any of the psychiatric scale scores (*p* > 0.05 for all). Although a negative correlation was observed between paternal age and stress factors (r = −0.171, *p* = 0.054), it did not reach statistical significance (Table 8).

Distribution of Psychiatric scale scores based on the presence of prior psychosocial symptoms is shown in Table 9. Adolescents with a history of psychosocial symptoms had significantly higher scores on the Anxiety Disorders Screening Scale (median = 36.0) compared to those without such a history (median = 26.5) (*p* = 0.027). The total scores on the Brief Symptom Inventory (BSI) were markedly higher among those with previous psychosocial symptoms (median = 70.5) than those without (median = 24.0), and this difference was statistically significant (*p* = 0.004) (Table 9).

Given the number of correlational analyses performed, these findings should be interpreted as exploratory, and the potential risk of Type I error inflation due to multiple testing should be considered.

Correlation analysis among scales applied to participants is shown in Table 10. There was a moderate positive correlation between the Anxiety Disorders Screening Scale and both the Stress Factors Scale (r = 0.375, *p* < 0.001) and the Trait Anxiety Inventory (r = 0.357, *p* < 0.001), as well as a strong correlation with the Brief Symptom Inventory (BSI) Total Score (r = 0.536, *p* < 0.001). The Trait Anxiety Inventory was also significantly correlated with the Stress Factors Scale (r = 0.344, *p* < 0.001), State Anxiety Inventory (r = 0.233, *p* = 0.008), and BSI Total Score (r = 0.389, *p* < 0.001). Additionally, the Stress Factors Scale was positively correlated with the BSI Total Score (r = 0.231, *p* = 0.009), and the Predisposing Factors Scale showed a weaker but significant correlation with the BSI Total Score (r = 0.199, *p* = 0.025) (Table 10).

The correlation analysis of the subscales of the BSI demonstrated statistically significant and strong positive associations across nearly all domains. The BSI total score was most strongly correlated with anxiety (r = 0.892), followed by obsessive–compulsive symptoms (r = 0.862), depression (r = 0.845), and additional symptoms (r = 0.853). Additionally, high inter-correlations were observed between several symptom domains: anxiety and depression (r = 0.802), depression and obsessive–compulsive symptoms (r = 0.735), anxiety and obsessive–compulsive symptoms (r = 0.718), and depression and paranoid ideation (r = 0.669) (Table 11).

The correlation analysis between the total score of the BSI and its global indices revealed strong and statistically significant relationships. The BSI total score was highly correlated with the Positive Symptom Total (r = 0.944, *p* < 0.001) and moderately correlated with the Symptom Distress Index (r = 0.691, *p* < 0.001). Additionally, a moderate positive correlation was found between the Positive Symptom Total and the Symptom Distress Index (r = 0.447, *p* < 0.001) (Table 12.).

## 4. Discussion

This study aimed to investigate the internalizing characteristics of adolescents presenting with non-cardiac chest pain (NCCP), with a specific focus on symptoms of anxiety and depression. The findings confirmed that NCCP in adolescents was strongly associated with increased emotional distress, especially anxiety-related symptoms, and that these symptoms frequently co-occurred with other psychiatric features such as somatization and obsessive–compulsive traits.

Our study emphasized the high prevalence of anxiety symptoms among adolescents with NCCP. Eliacik et al. found that children with recurrent somatic complaints, including chest pain, exhibited elevated levels of both anxiety and depression when compared to healthy peers [7]. Herge et al. highlighted that adolescents with unexplained physical complaints often demonstrated higher internalizing scores, suggesting a strong emotional basis for their symptoms [8]. These findings aligned with our results, wherein a substantial portion of participants exhibited anxiety scores exceeding clinical thresholds. In addition, studies by Voepel-Lewis et al. and Bohman et al. have noted similar psychosomatic presentations linked with underlying anxiety disorders in youth [10,19].

However, our study differed from previous research in several ways. While prior studies largely focused on the presence of anxiety or depressive symptoms in youth with somatic complaints, we extended the analysis to include a broader range of internalizing symptoms using multiple validated psychiatric scales. We also evaluated specific sociodemographic influences such as maternal and paternal age, parental education level, and prior psychosocial symptom history—variables that had not been thoroughly explored in the earlier literature. Unlike previous studies, we found that maternal age was significantly associated with lower stress levels in adolescents, while maternal education level was not—a nuance not explicitly reported in earlier findings [8,11,16,17,18].

A notable finding was the significant gender difference in symptom presentation. Girls reported higher scores on the Anxiety Disorders Screening Scale, the Stress Factors Scale, and the Brief Symptom Inventory (BSI), indicating that females in this population were more vulnerable to internalizing psychological distress. Cosma et al. concluded that adolescent girls were generally more likely to internalize stress and present with somatic symptoms [13]. The elevated anxiety and symptom scores among girls may have been attributed to hormonal, social, or cognitive–emotional factors that amplified stress responses. Rapee et al. reported greater emotional reactivity and anxiety sensitivity in adolescent females [12].

As a secondary and exploratory finding, maternal age was negatively correlated with adolescents’ perceived stress levels. This may have suggested that older mothers, potentially due to greater parenting experience or emotional maturity, provided more stable and supportive environments, thereby buffering adolescents from stress. Albanese et al. reported similar results, indicating that maternal psychosocial characteristics played a more decisive role in a child’s emotional well-being than paternal attributes [14]. In contrast, no significant correlations were observed between paternal age and any of the psychiatric indicators, underlining the unique role of maternal factors in adolescent psychological adjustment [15]. Li et al. emphasized that maternal psychological flexibility has a stronger association with adolescent emotional outcomes than paternal involvement [23].

The presence of prior psychosocial symptoms (such as previous episodes of anxiety, behavioral issues, or family stress) was associated with significantly higher anxiety and BSI scores. This finding was in line with the literature, where early emotional disturbances were known to predispose individuals to recurrent somatic complaints. Pate et al. demonstrated that children with a history of psychological distress were more likely to report persistent pain and functional impairment [22]. While previous studies noted the presence of psychosocial vulnerabilities, our study provided quantitative evidence of their relationship with specific symptom severity. Noel et al. showed that adverse childhood experiences significantly elevated the risk of somatic symptom disorders in adolescence [21].

Correlation analyses provided further insight into the overlapping nature of internalizing symptoms. Anxiety, trait anxiety, and depression were all significantly associated with the BSI total score, reflecting the centrality of these dimensions in adolescents with NCCP. Particularly, the strong correlations between anxiety and depression (r = 0.802), as well as between anxiety and obsessive–compulsive symptoms (r = 0.718), suggested a comorbid cluster of emotional dysregulations. Kallesoe et al. reported that adolescents with functional somatic symptoms frequently experienced multiple, overlapping internalizing disorders [9]. However, our study uniquely quantified these associations using comprehensive psychometric data.

Furthermore, analysis of the subscales within the BSI revealed consistent inter-correlations, particularly between anxiety, depression, somatization, and obsessive–compulsive domains. The BSI total score was most strongly predicted by anxiety (r = 0.892), supporting the hypothesis that anxiety was the most dominant psychological feature in adolescents with NCCP. The relationship between positive symptom count and total psychological burden (r = 0.944) indicated that both symptom intensity and breadth were relevant for clinical evaluation. This multi-dimensional insight into symptom clustering enhanced the interpretability of previous single-scale studies.

Another important implication was the lack of significant associations between sociodemographic variables such as education level or parental employment status and psychiatric symptom severity. This suggested that emotional distress in adolescents with NCCP was not primarily driven by socioeconomic status, but rather by personal, psychological, and familial dynamics. O’Connell et al. emphasized that adolescents with unexplained physical symptoms benefited more from psychological support than from repeated medical testing, which often yielded no conclusive findings [20]. Likewise, the findings of Nelson et al. [13] and Friedrichsdorf et al. also underscored the importance of psychological evaluation in recurrent somatic pain presentations [34,35].

From a clinical perspective, the present findings highlight the importance of adopting a more integrated approach to adolescents presenting with non-cardiac chest pain. The high burden of anxiety-related symptoms and global psychological distress observed in this population suggests that NCCP should not be viewed solely as a benign exclusion diagnosis following cardiac evaluation, but rather as a potential marker of underlying emotional distress. Routine incorporation of brief psychological screening tools in pediatric cardiology and general pediatric outpatient settings may facilitate early identification of adolescents at risk for internalizing disorders.

The observed gender differences further emphasize the need for heightened clinical awareness in female adolescents, who may be more vulnerable to internalizing symptomatology and somatic expression of stress. Early recognition of these patterns may help clinicians avoid repeated diagnostic testing, reduce healthcare utilization, and guide timely referral to mental health services when appropriate. In addition, the association between prior psychosocial symptoms and greater psychological distress underscores the value of taking a focused psychosocial history in adolescents presenting with chest pain.

Notably, the inverse relationship between maternal age and perceived stress suggests that family-related contextual factors may play a buffering role in adolescent stress regulation. This finding supports the inclusion of family-oriented perspectives in clinical assessment and highlights the potential benefit of psychoeducation and family-based interventions aimed at improving emotional coping strategies.

This study had some limitations. First, the cross-sectional design precluded any causal inferences between psychological symptoms and non-cardiac chest pain. Longitudinal studies would be more appropriate to evaluate temporal relationships and causality. Second, the sample size, although adequate for statistical analysis, was drawn from a single-center pediatric clinic, which may limit the generalizability of the findings to broader or more diverse populations. Third, all psychiatric assessments were based on self-report measures, which can be subject to social desirability bias and may not fully capture the complexity of psychiatric conditions. Future research could benefit from including structured clinical interviews or multi-informant reports. Lastly, while this study included multiple validated psychiatric scales, it did not account for other possible influencing factors such as peer relationships, academic performance, or recent life events, which may also contribute to internalizing symptoms in adolescents. Another limitation of this study is the absence of age-stratified analyses distinguishing early and late adolescence. Although stressors and emotional responses may vary across developmental stages, the present study was not powered to conduct reliable subgroup analyses. Future studies with larger samples should examine age-specific patterns of stress and internalizing symptoms to better capture developmental differences within adolescent populations.

## 5. Conclusions

In conclusion, adolescents presenting with non-cardiac chest pain exhibit a substantial burden of internalizing psychological symptoms, particularly anxiety and depression. Female gender and a history of psychosocial difficulties were identified as important risk factors, while maternal age appeared to have a potential buffering effect on perceived stress. These findings suggest that non-cardiac chest pain in adolescence may serve as a clinically relevant indicator of underlying emotional distress.

From a practical standpoint, integrating psychological screening into the routine evaluation of adolescents with chest pain may improve early detection of internalizing disorders, support timely mental health referral, and reduce unnecessary medical investigations. A multidisciplinary, biopsychosocial approach involving pediatricians, cardiologists, and mental health professionals may therefore enhance both clinical outcomes and healthcare efficiency in this population.

## Figures and Tables

**Table 1:** Demographic characteristics of the study group.

Variables	n (%)
Educational Level	
Primary School	16 (12.5%)
Middle School	62 (48.4%)
High School	50 (39.1%)
Mother’s Marital Status	
Married	112 (87.5%)
Divorced	16 (12.5%)
Mother’s Education Level	
Literate	1 (0.8%)
Primary School	64 (50.0%)
Middle School	23 (18.0%)
High School	22 (17.1%)
University	18 (14.1%)
Mother’s Employment Status	
Housewife	83 (64.8%)
Employed	45 (35.2%)
Father’s Education Level	
Illiterate	1 (0.8%)
Literate	1 (0.8%)
Primary School	48 (37.9%)
Middle School	27 (21.3%)
High School	23 (18.2%)
University	27 (21.3%)
Father’s Occupation	
Worker	29 (23.0%)
Civil Servant	24 (19.0%)
Trader	23 (18.3%)
Self-employed	50 (39.7%)

**Table 2:** Percentage of participants’ responses to the Stress Factors Scale.

Variables	n (%)
Fear of Being Questioned by Parents	32 (25.0)
Fear of Getting an Injection	53 (41.4)
Fear of Seeing Blood	28 (21.9)
Fear of Talking to Strangers	41 (32.0)
Fear of Being Forced to Share Personal Information	44 (34.4)
Fear of Being Separated from Mother or Father	69 (53.9)
Fear of Undressing During Doctor Examination	42 (32.8)
Fear of Being Hospitalized	38 (29.7)
Fear of Experiencing Pain	73 (57.0)

**Table 3:** Percentage of participants’ responses to Predisposing Factors Scale.

Variables	n (%)
Moving House in the Last Six Months	18 (14.1)
Having a Very Close Friend	106 (82.8)
Skipping School, Fighting or Committing a Disciplinary Offense	20 (15.6)
New Sibling Born in the Family Within the Last Year	11 (8.6)
Changing School in the Last Month	22 (17.2)
Changing Class in the Last Six Months	19 (14.8)
Death in the Family in the Last Two Years	14 (10.9)
Divorce in the Family Within the Last Two Years	10 (7.8)

**Table 4:** Scores received by participants from Psychiatric Scales.

Variables	Mean ± SD	Median (Min–Max)
Anxiety Disorders Screening Scale	29.5 ± 12.1	27.0 (10.0–82.0)
State Anxiety Inventory	42.8 ± 7.0	43.0 (18.0–61.0)
Trait Anxiety Inventory	44.9 ± 6.4	45.0 (33.0–67.0)
Brief Symptom Inventory	37.6 ± 34.6	25.0 (0.0–152.0)

**Table 5:** Distribution of Psychiatric Scale Scores according to maternal education level.

Variables	Primary School and Below (n = 88)		High School and Above (n = 40)		
	Mean ± SD	Median (Min–Max)	Mean ± SD	Median (Min–Max)	*p* Value
Stress Factors	3.5 ± 2.3	3.0 (0.0–8.0)	2.8 ± 2.2	3.0 (0.0–7.0)	0.148
Predisposing Factors	1.8 ± 1.4	1.0 (0.0–7.0)	1.5 ± 0.9	1.0 (0.5–4.0)	0.455
Anxiety Disorders Screening Scale	28.5 ± 9.9	27.0 (10–64)	30.5 ± 14.0	26.0 (10–82)	0.445
State Anxiety Inventory	42.8 ± 7.2	43.0 (28.0–61.0)	42.9 ± 6.9	43.0 (30.0–58.0)	0.567
Trait Anxiety Inventory	44.7 ± 6.1	44.0 (33.0–58.0)	45.7 ± 7.4	45.0 (33.0–67.0)	0.431
Brief Symptom Inventory (BSI)—Total	37.9 ± 35.9	25.0 (0.0–152.0)	36.9 ± 31.9	29.0 (1.0–131.0)	0.960

**Table 6:** Distribution of Psychiatric Scale scores by gender.

Variables	Girls (n = 73)		Boys (n = 55)		
	Mean ± SD	Median (Min–Max)	Mean ± SD	Median (min–MAX)	*p* Value
Stress Factors	3.86 ± 2.0	4.0 (0.0–8.0)	2.50 ± 2.0	2.0 (0.0–8.0)	0.001 *
Predisposing Factors	1.80 ± 1.3	1.0 (0.0–7.0)	1.60 ± 1.2	1.0 (0.0–6.0)	0.277
Anxiety Disorders Screening Scale	32.79 ± 13.6	29.0 (10–82)	25.31 ± 7.9	24.0 (12–49)	0.000 *
State Anxiety Inventory	42.9 ± 6.8	43.0 (30.0–61.0)	42.8 ± 7.5	43.0 (18.0–57.0)	0.645
Trait Anxiety Inventory	45.9 ± 6.8	45.0 (33.0–67.0)	43.8 ± 5.5	43.0 (34.0–57.0)	0.075
Brief Symptom Inventory—Total	45.9 ± 31.2	33.0 (0.0–152.0)	26.9 ± 27.9	18.0 (0.0–131.0)	0.001 *

*: Mann–Whitney U test.

**Table 7:** Correlation between maternal age and Psychiatric Scales.

Variables	Correlation Coefficient (r)	*p*-Value
Stress Factors	−0.259	0.003
Predisposing Factors	−0.069	0.440
Anxiety Disorders Screening Scale	0.044	0.625
State Anxiety	−0.101	0.258
Trait Anxiety	−0.111	0.214
BSI Total Score	0.106	0.238

Spearman correlation analysis was used.

**Table 8:** Correlation between paternal age and Psychiatric Scales.

Variables	Correlation Coefficient (r)	*p*-Value
Stress Factors	−0.171	0.054
Predisposing Factors	−0.128	0.149
Anxiety Disorders Screening Scale	0.014	0.875
State Anxiety	−0.003	0.973
Trait Anxiety	−0.008	0.925
BSI Total Score	0.120	0.182

**Table 9:** Distribution of Psychiatric Scale scores based on the presence of prior psychosocial symptoms.

Variables	Symptoms Present (n = 8)	Symptoms Absent (n = 120)	*p*-Value
	Median (Min–Max)		
Stress Factors	4.0 (0.0–5.0)	3.0 (0.0–8.0)	0.736
Predisposing Factors	1.5 (0.0–7.0)	1.0 (0.0–7.0)	0.770
Anxiety Disorders Screening Scale	36.0 (23–82)	26.5 (10.0–64.0)	0.027
State Anxiety Inventory	43.0 (38–60)	43.0 (18.0–61.0)	0.415
Trait Anxiety Inventory	48.0 (42–58)	44.0 (33.0–67.0)	0.058
BSI Total Score	70.5 (27–152)	24.0 (0.0–144.0)	0.004

**Table 10:** Correlation analysis among scales applied to participants.

Variable	1	2	3	4	5	6
1. Stress Factors	—					
2. Predisposing Factors	0.11	—				
3. Anxiety Screening	0.38 **	0.14	—			
4. State Anxiety	0.05	0.07	0.02	—		
5. Trait Anxiety	0.34 **	0.05	0.36 **	0.23 **	—	
6. BSI Total Score	0.23 **	0.20 *	0.54 **	−0.09	0.39 **	—

*: *p* < 0.05, **: *p* < 0.01. BSI: Brief Symptom Inventory.

**Table 11:** Correlation analysis between subscales of the Brief Symptom Inventory (BSI).

Subscales	SOM	OCD	INT	DEP	ANX	PAR	PSY	ADD	TOTAL
Somatization	1								
Obsessive–Compulsive	0.636 **	1							
Interpersonal Sens.	0.553 **	0.616 **	1						
Depression	0.617 **	0.735 **	0.623 **	1					
Anxiety	0.708 **	0.718 **	0.606 **	0.802 **	1				
Paranoid Ideation	0.594 **	0.706 **	0.641 **	0.669 **	0.703 **	1			
Psychoticism	0.543 **	0.668 **	0.499 **	0.686 **	0.671 **	0.682 **	1		
Additional Items	0.695 **	0.650 **	0.646 **	0.680 **	0.740 **	0.683 **	0.617 **	1	
BSI Total Score	0.819 **	0.862 **	0.749 **	0.845 **	0.892 **	0.830 **	0.769 **	0.853 **	1

SOM = Somatization; OCD = Obsessive–Compulsive symptoms; INT = Interpersonal Sensitivity; DEP = Depression; ANX = Anxiety; PAR = Paranoid Ideation; PSY = Psychoticism; ADD = Additional Items. All correlations are significant at the *p* < 0.001 level (Spearman correlation).

**Table 12:** Correlation Between BSI Total Score and BSI Index Scores.

Variable	1	2	3
1. BSI Total Score	—		
2. Positive Symptom Total	0.94 **	—	
3. Symptom Distress Index	0.69 **	0.45 **	—

**: *p* < 0.01. BSI: Brief Symptom Inventory.

## Data Availability

Data is available upon request to the corresponding author.

## Abbreviations

NCCP	Non-Cardiac Chest Pain
CDI	Children’s Depression Inventory
STAI-C	State–Trait Anxiety Inventory for Children
STAI-I	State Anxiety Inventory
STAI-II	Trait Anxiety Inventory
SCARED	Screen for Child Anxiety Related Emotional Disorders
BSI	Brief Symptom Inventory
SSAS-C	Social Support Appraisals Scale for Children
OCD	Obsessive–Compulsive Disorder
SOM	Somatization
INT	Interpersonal Sensitivity
DEP	Depression
ANX	Anxiety
PAR	Paranoid Ideation
PSY	Psychoticism
ADD	Additional Items
SD	Standard Deviation

## References

[1] Saleeb S.F., McLaughlin S.R., Graham D.A., Friedman K.G., Fulton D.R. (2018). Resource reduction in pediatric chest pain: Standardized clinical assessment and management plan. Congenit. Heart Dis..

[2] Huang X., Jia N., Zhang Y., Hao Y., Xiao F., Sun C., Cui X., Wang F. (2024). Effect of cognitive-behavior therapy for children with functional abdominal pain: A meta-analysis. BMC Gastroenterol..

[3] Khan B., Avan B.I. (2020). Behavioral problems in preadolescence: Does gender matter?. Psych. J..

[4] Cai L., Yuan W. (2025). Analysis of the influence of emotional factors on the efficacy and prognosis of endoscopic treatment of functional abdominal pain in children. Medicine.

[5] Cole M.A.T., Qu’d D., Wild M.G., Russell A.C., Caillet A.R., Stone A.L. (2020). “My Body Hates Me”: A Qualitative Analysis of the Experience of Functional Nausea in Adolescent Girls and Their Mothers. Children.

[6] Munker L., Rimvall M.K., Frostholm L., Ornbol E., Wellnitz K.B., Jeppesen P., Maria Rosmalen J.G., Rask C.U. (2024). Exploring the course of functional somatic symptoms (FSS) from pre- to late adolescence and associated internalizing psychopathology—An observational cohort-study. BMC Psychiatry.

[7] Eliacik K., Bolat N., Kanik A., Malas N., Demircan T., Hortu H., Ozyurt G., Orbatu D., Alaygut D., Guven B. (2020). Adolescents with unexplained chest pain reported depression and impaired emotional and social functioning. Acta Paediatr..

[8] Herge W.M., La Greca A.M., Chan S.F. (2016). Adolescent Peer Victimization and Physical Health Problems. J. Pediatr. Psychol..

[9] Kallesoe K.H., Rimvall M.K., Schroder A., Jensen J.S., Wicksell R.K., Rask C.U. (2021). Adolescents with functional somatic syndromes: Symptom profiles, illness perception, illness worry and attachment orientation. J. Psychosom. Res..

[10] Bohman H., Laftman S.B., Cleland N., Lundberg M., Paaren A., Jonsson U. (2018). Somatic symptoms in adolescence as a predictor of severe mental illness in adulthood: A long-term community-based follow-up study. Child Adolesc. Psychiatry Ment. Health.

[11] Kolaitis G., van der Ende J., Zaravinos-Tsakos F., White T., Derks I., Verhulst F., Tiemeier H. (2022). The occurrence of internalizing problems and chronic pain symptoms in early childhood: What comes first?. Eur. Child Adolesc. Psychiatry.

[12] Rapee R.M., Oar E.L., Johnco C.J., Forbes M.K., Fardouly J., Magson N.R., Richardson C.E. (2019). Adolescent development and risk for the onset of social-emotional disorders: A review and conceptual model. Behav. Res. Ther..

[13] Cosma A., Kolto A., Badura P., Winkler P., Kalman M. (2021). Time trends in adolescent mental wellbeing in the Czech Republic between 2002 and 2018: Gender, age and socioeconomic differences. Cent. Eur. J. Public Health.

[14] Albanese A.M., Russo G.R., Geller P.A. (2019). The role of parental self-efficacy in parent and child well-being: A systematic review of associated outcomes. Child Care Health Dev..

[15] Koutra K., Paschalidou A., Roumeliotaki T., Triliva S. (2023). Main and interactive retrospective associations between parental rearing behavior and psychological adjustment in young adulthood. Curr. Psychol..

[16] Feng Y., Zhang S., Liao X., Jia Y., Yang Y., Zhang W. (2024). Association between bullying victimization and mental health problems among Chinese left-behind children: A cross-sectional study from the adolescence mental health promotion cohort. Front. Psychiatry.

[17] Heller P., Morosan L., Badoud D., Laubscher M., Jimenez Olariaga L., Debbane M., Wolff H., Baggio S. (2021). Prevalence Rates and Evolution of Psychiatric Disorders Among Incarcerated Youths in Comparison with Non-incarcerated Youths. Front. Psychiatry.

[18] Lee K.S., Vaillancourt T. (2019). Unraveling the long-term links among adolescent peer victimization and somatic symptoms: A 5-year multi-informant cohort study. J. Appl. Biobehav. Res..

[19] Voepel-Lewis T., Seng J.S., Chen B., Scott E.L. (2021). A High Psychological and Somatic Symptom Profile and Family Health Factors Predict New or Persistent Pain During Early Adolescence. Clin. J. Pain.

[20] O’Connell C., Shafran R., Bennett S. (2020). A systematic review of randomised controlled trials using psychological interventions for children and adolescents with medically unexplained symptoms: A focus on mental health outcomes. Clin. Child Psychol. Psychiatry.

[21] Herzog J.I., Schmahl C. (2018). Adverse Childhood Experiences and the Consequences on Neurobiological, Psychosocial, and Somatic Conditions Across the Lifespan. Front. Psychiatry.

[22] Pate J.W., Hancock M.J., Hush J.M., Gray K., Pounder M., Pacey V. (2020). Prognostic factors for pain and functional disability in children and adolescents with persisting pain: A systematic review and meta-analysis. Eur. J. Pain.

[23] Li M., Chen X., Gong H., Wang W., Ji W., Liang S. (2022). Relationship between paternal adult attachment and adolescent anxiety: The chain-mediating effect of paternal psychological flexibility and father-adolescent attachment. Int. J. Psychol..

[24] Kovacs M. (1985). The Children’s Depression, Inventory (CDI). Psychopharmacol. Bull..

[25] Oy B. (1991). Cocuklar icin Depresyon Olcegi gecerlik ve guvenirlik calismasi. Turk. Psikiyatr. Derg..

[26] Spielberger C.D., Gorsuch R.L., Lushene R.E. (1983). Manual for the State-Trait Anxiety Inventory.

[27] Özusta H.Ş. (1995). Çocuklar için durumlu-sürekli kaygı envanteri. Uyarlama, geçerlik ve güvenirlik çalışması. Türk. Psikol. Derg..

[28] Birmaher B., Khetarpal S., Brent D., Cully M., Balach L., Kaufman J., Neer S.M. (1997). The Screen for Child Anxiety Related Emotional Disorders (SCARED): Scale construction and psychometric characteristics. J. Am. Acad. Child Adolesc. Psychiatry.

[29] Yalin Sapmaz S., Ozek Erkuran H., Ergin D., Ozturk M., Sen Celasin N., Karaarslan D., Aydemir O. (2018). Validity and reliability of the Turkish version of the DSM-5 Generalized Anxiety Disorder Severity Scale for children aged 11–17 years. Turk. J. Med. Sci..

[30] Derogatis L.R., Melisaratos N. (1983). The Brief Symptom Inventory: An introductory report. Psychol. Med..

[31] Şahin H.N., Uğurtaş S., Batigün D.A. (2002). Kısa Semptom Envanteri (KSE): Ergenler için kullanımının geçerlik, güvenilirlik ve faktör yapısı. Türk. Psikiyatr. Derg..

[32] Dubow E.F., Ullman D.G. (1989). Assessing social support in elementary school children: The survey of children’s social support. J. Clin. Child Psychol..

[33] Gökler I. (2007). Çocuk ve ergenler için sosyal destek değerlendirme ölçeği Türkçe formunun uyarlama çalışması: Faktör yapısı, geçerlik ve güvenirliği. Çocuk Ve Gençlik Ruh Sağlığı Derg..

[34] Nelson S., Agoston M., Kovar-Gough I., Cunningham N. (2022). A Scoping Review and Proposed Framework for Coping in Youth with a History of Psychological Trauma and Chronic Pain. J. Pediatr. Psychol..

[35] Friedrichsdorf S.J., Giordano J., Desai Dakoji K., Warmuth A., Daughtry C., Schulz C.A. (2016). Chronic Pain in Children and Adolescents: Diagnosis and Treatment of Primary Pain Disorders in Head, Abdomen, Muscles and Joints. Children.

